# The effects of age, mature oocyte number, and cycle number on cumulative live birth rates after planned oocyte cryopreservation

**DOI:** 10.1007/s10815-024-03175-w

**Published:** 2024-07-02

**Authors:** Sarah Druckenmiller Cascante, James A. Grifo, Frederick Licciardi, Carlos M. Parra, Amelia Kelly, Alan S. Berkeley

**Affiliations:** New York University Langone Fertility Center, 159 East 53rd Street, 3rd Floor, 10022 New York, NY USA

**Keywords:** Egg freezing, Oocyte cryopreservation, Oocyte thaw, Egg thaw, Fertility preservation, Assisted reproductive technology

## Abstract

**Purpose:**

To examine the effects of age, mature oocyte number, and cycle number on cumulative live birth rates after planned oocyte cryopreservation (OC), with the goal of developing a patient counselling tool.

**Methods:**

We performed a retrospective cohort study of all patients with ≥ 1 autologous oocyte thaw at our university-affiliated fertility center before 12/31/2023. Patients were included if they (1) had a live birth or ongoing pregnancy > 12 weeks from OC, or (2) used all oocytes and euploid/untested embryos from OC. Primary outcome was cumulative live birth / ongoing pregnancy rate (CLBR).

**Results:**

527 patients with 1 OC cycle, 149 patients with 2 OC cycles, and 55 patients with ≥ 3 OC cycles were included. Overall CLBR was 43%. CLBR was > 70% among patients who thawed ≥ 20 mature oocytes that were cryopreserved at age < 38 years. Multiple logistic regression showed that age at first OC and total number of mature oocytes thawed independently predicted CLBR, but number of OC cycles did not.

**Conclusion:**

Patients must be counselled that younger age at OC and more mature oocytes improve CLBR. However, additional OC cycles do not independently improve CLBR. Our results can help patients decide whether to pursue additional OC cycles to obtain more oocytes.

## Introduction

Planned oocyte cryopreservation (OC) is now widely accepted as a fertility preservation method for women facing age-related fertility decline. This technology can result in pregnancy rates comparable with fresh in vitro fertilization (IVF) [1–7] and appears safe for offspring [2, 8]. A recent report from our center, which reviewed 543 planned OC patients who thawed autologous oocytes, demonstrated that the majority of patients who were < 38 years (y) at OC or who thawed ≥ 20 metaphase II oocytes (M2s) had a live birth from OC [1]. As expected, we also showed that age at OC and number of M2s thawed were predictive of live birth. Lastly, we demonstrated that patients who underwent two OC cycles had a higher likelihood of live birth than patients who underwent one OC cycle, suggesting that a second OC cycle increases the chance of live birth.

However, it is still unclear whether additional OC cycles independently increase the chance of live birth, or whether the benefit of additional OC cycles can be explained entirely by the increased number of oocytes. Thus, the optimal number of OC cycles and optimal number of oocytes to cryopreserve remain unknown. Patients and physicians often seek guidance from Goldman et al.’s predictive model, which uses data extrapolated from IVF patients with normal ovarian reserve and oocyte donors [9]; however, this model does not account for the number of OC cycles and its correctness is unknown. Another model by Maslow et al. used data from 1799 OC cycles at a single center and estimated live birth rates from previously published data to extrapolate age-based oocyte thresholds for 50%, 60%, and 70% estimated live birth rates [10]. However, the estimated live birth rates were also derived in part from patients with infertility diagnoses and oocyte donors, rather than from planned OC patients alone. Patients yearn for more information about thaw outcomes from actual planned OC patients so they can make informed decisions about the role of OC in achieving their family building goals.

After completing one OC cycle, patients often inquire about: (1) the chance of live birth from their cryopreserved oocytes, and (2) how much additional OC cycles will increase their chance of live birth. However, these questions remain difficult to answer due to the dearth of planned OC outcome data. Therefore, our aim was to examine cumulative live birth rate (CLBR) based on age, total number of M2s thawed, and the total number of OC cycles, with the goal of developing a patient counselling tool.

## Materials and Methods

### Design

With Institutional Review Board approval (number S13-00389), we performed a retrospective cohort study of all patients who underwent OC followed by autologous oocyte thaw/warming (“thaw” will be used for consistency) at our center prior to December 31, 2023. Transfers from resultant embryos were included if they occurred before the same date.

### Subjects

All patients who underwent at least one autologous oocyte thaw at our center during the study period were reviewed. Patients were included if they: (1) had at least one live birth or ongoing pregnancy > 12 weeks from OC, or (2) used all oocytes and euploid and untested embryos from OC. Patients were excluded if: (1) they had remaining cryopreserved oocytes or euploid/untested embryos from OC, but no live birth or ongoing pregnancy > 12 weeks from OC, (2) they had no live birth, but had a transfer of embryos created from OC after December 31, 2023, (3) OC was performed for a medical indication (e.g. fertility preservation prior to gonadotoxic therapy or gender-affirming treatment), due to a natural disaster (Hurricane Sandy), due to lack of sperm, or in combination with embryos, (4) they had a cancer diagnosis or planned to use a gestational carrier, or (5) pregnancy outcome was unknown. Of note, we included patients who underwent OC as part of a research protocol where the intent was to thaw oocytes within the following six months.

### Data Collection & Outcomes

Information regarding OC, oocyte thaw, and embryo transfer cycles were obtained from electronic medical records. Collected OC cycle data included: cryopreservation location (our facility, an outside facility, or both); patient age; number of total oocytes and M2 oocytes cryopreserved; and cryopreservation method (slow freezing vs. vitrification vs. both methods). Collected oocyte thaw cycle data included: number of total oocytes and M2 oocytes thawed and surviving thaw; number of usable embryos (defined as: embryos for preimplantation genetic testing (PGT), cryopreservation, or fresh transfer). Collected transfer data included: pregnancy and live birth outcomes. We also determined whether patients had remaining oocytes or euploid/untested embryos from OC in storage at our center or that were transported to another center, donated, or discarded as of December 31, 2023.

The primary outcome was CLBR, and was defined per patient. CLBR included patients who had a live birth or ongoing pregnancy. All ongoing pregnancies were > 12 weeks at data collection. Patients were stratified by age at first OC, total number of M2s thawed, and total number of OC cycles.

### OC, Thawing, and Embryo Transfer

Ovarian stimulation protocols were chosen by the treating physician based on the patient’s ovarian reserve and age. From 2004 to 2015, all retrieved M2s and metaphase I oocytes (M1s) were cryopreserved; however, after 2015, M1s were only cryopreserved if less than 15 M2s were obtained from the same OC cycle. Oocytes were cryopreserved with slow freezing or vitrification using previously described techniques [5]. Vitrification was used for all OC cycles performed after July 2011. During our center’s conversion from slow freezing to vitrification, a combination of both OC methods was often used to cryopreserve oocytes from a single retrieval. For this reason, some patients in this study had some oocytes that were slow frozen and some oocytes that were vitrified (from a single OC cycle and/or from multiple different OC cycles).

Oocytes were thawed using previously described techniques [5], and intracytoplasmic sperm injection was used for fertilization. Embryos were cultured until transfer (days 3–7 based on physician instructions), trophectoderm biopsy for PGT, or cryopreservation on days 5–7. In order to be biopsied for PGT, embryos were required to have a Gardner’s score of A or B for the inner cell mass or for the trophectoderm. PGT was performed with array comparative genomic hybridization or next generation sequencing based on what technology was standard in our laboratory at the time of thaw. Endometrial preparation for embryo transfer was achieved using previously described techniques [1]. Typically, embryos with euploid status and/or superior morphology were given preference for transfer.

### Statistical Analysis

The Kolmogorov-Smirnoff test was used to assess continuous variables for normality, and these variables were found to be non-parametric. Chi-square tests were used to analyze categorical variables. An alpha error of 0.05 was deemed significant. Results are reported as percentages or medians with interquartile ranges (IQRs).

Lastly, multiple logistic regression was used for modeling and adjustment of covariates (age at first OC, total number of M2s thawed, and total number of OC cycles) to evaluate the outcome of cumulative live birth / ongoing pregnancy. Multiple regression was performed using stepwise addition of parameters that satisfied the Akaike information criterion. 95% confidence intervals were assembled using likelihood intervals (e-fold reduction in maximum likelihood allowing all other parameters to adjust freely). The purpose of this model was to facilitate patient counseling.

## Results

A total of 731 patients were included. 527 patients underwent 1 OC cycle, 149 patients underwent 2 OC cycles, 37 patients underwent 3 OC cycles, 10 patients underwent 4 OC cycles, 4 patients underwent 5 OC cycles, 2 patients underwent 6 OC cycles, and 2 patients underwent 8 OC cycles. The median number of OC cycles was 1 and the mean number of OC cycles was 1.4. OC was performed at our facility for 89% of patients, an outside facility for 9% of patients, and both for 2% of patients.

Ninety-six percent of patients (*n* = 701) did not have a prior infertility diagnosis; 4% (*n* = 30) of patients underwent OC as part of a research protocol where the intent was to thaw oocytes within the following six months. Patients who underwent OC as part of a research protocol were ≤ 37 years at the time of OC, had normal baseline follicle stimulating hormone levels, and may have had an infertility diagnosis (etiologies: unexplained, polycystic ovarian syndrome, tubal disease).

Table 1 shows the age distribution of our cohort at the time of first OC. The median age at first OC was 38y (IQR: 36—39). The median number of M2s cryopreserved in the first OC cycle was 10 (IQR: 6—16), and the median total number of M2s cryopreserved was 13 (IQR: 8—20). The cryopreservation method was vitrification alone for 78% of patients, slow freezing alone for 4% of patients, a combination of both vitrification and slow freezing for 18% of patients, and unknown for < 1% of patients.

**Table 1:** Age distribution of cohort at time of first cryopreservation

Age at first cryopreservation (years)	Number of patients	Percentage of cohort
< 35	100	13.7%
35–37	261	35.7%
38–40	283	38.7%
> 41	87	11.9%

The median age at first thaw was 42 years (IQR: 40 – 44), and the median time between first OC and first thaw was 4.6 years (IQR: 3.0 – 6.1 years; maximum: 13.2 years). The median total number of M2s thawed was 12 (IQR: 8 – 18.5) and the median total number M2s that survived thaw was 10 (IQR: 5—15). Patients had a median of 3 usable embryos (IQR 1 – 5) from OC. Among patients with ≥ 1 useable embryo, 73% (*n* = 449/618) used PGT-A to test ≥ 1 embryo. The vast majority of embryo transfers (92%) were performed on day 5 or 6; however, 4% were performed on day 3 or 4 and 4% were performed on day 7. The CLBR among all patients was 43% (*n* = 313/731).

CLBRs did not differ based on whether patients underwent 1, 2, or ≥ 3 OC cycles (*p* = 0.20). The CLBR was 41% (*n* = 215/527) among patients who underwent 1 OC cycle, 48% (*n* = 71/149) among patients who underwent 2 OC cycles, and 49% (*n* = 27/55) among patients who underwent ≥ 3 OC cycles.

**Fig. 1:**
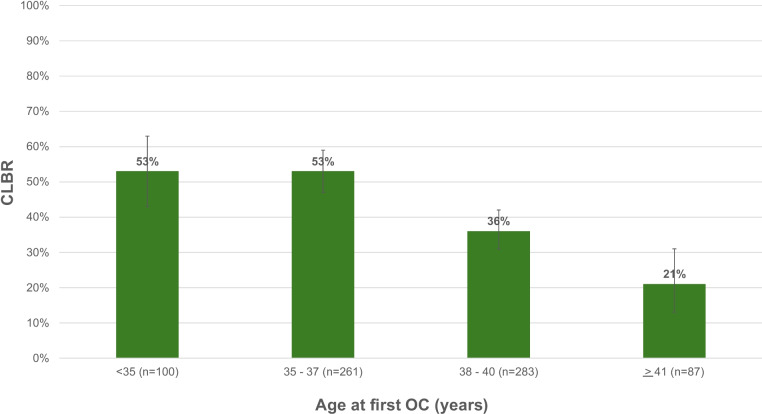
Cumulative live birth / ongoing pregnancy rates (CLBRs) per patient based on age at first oocyte cryopreservation (OC)

**Fig. 2:**
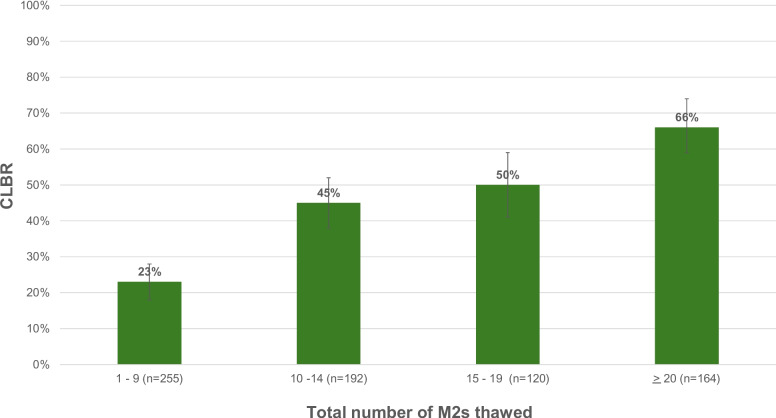
Cumulative live birth / ongoing pregnancy rates (CLBRs) per patient based on total number of metaphase II oocytes (M2s) thawed

CLBR differed among patients of different ages (*p* < 0.01) and among patients who thawed different numbers of M2s (*p* < 0.01). Figure 1. shows CLBR by age, and Fig. 2 shows CLBR by total number of M2s thawed. Table 2 shows CLBR when patients were stratified by age at first cryopreservation and total number of M2s thawed.

**Table 2:** Cumulative live birth / ongoing pregnancy rates (CLBRs) per patient based on age at first cryopreservation and total number of metaphase II oocytes (M2s) thawed

Age at first cryopreservation (years)	1 – 9 M2s thawed *(n* = 255)	10 – 14 M2s thawed (*n* = 192)	15 – 19 M2s thawed (*n* = 120)	> 20 M2s thawed (*n* = 164)
< 35 (*n* = 100)	33% [13 – 59%] (*n* = 6/18)	35% [17 – 56%] (*n* = 9/26)	64% [43 – 82%] (*n* = 16/25)	71% [52 – 86%] (*n* = 22/31)
35–37 (*n* = 261)	32% [21 – 44%] (*n* = 22/69)	53% [41 – 64%] (*n* = 41/78)	45% [29 – 62%] (*n* = 18/40)	78% [67 – 87%] (*n* = 58/74)
38–40 (*n* = 283)	21% [14 – 29%] (*n* = 25/120)	44% [32 – 56%] (*n* = 31/71)	49% [34 – 64%] (*n* = 23/47)	53% [38 – 68%] (*n* = 24/45)
≥ 41 (*n* = 87)	10% [3 – 23%] (*n* = 5/48)	29% [10 – 56%] (*n* = 5/17)	38% [9 – 76%] (*n* = 3/8)	36% [13 – 65%] (*n* = 5/14)

Note: 95% confidence intervals are shown in square brackets

Multiple logistic regression shows that age at first OC (B = -0.14; 95% confidence interval [CI] = -0.19 to -0.10), and total number of M2s thawed (B = 0.07; 95% CI = 0.06 to 0.08) independently predicted CLBR. The number of OC cycles was not included in the multiple regression model since it was not a significant predictor of live birth (failed to exceed the Akaike information criterion and its coefficient was not significantly different than zero when included in the model). The variance inflation factors were as follows: age at first OC = 1.04, total number of M2s thawed = 1.23, number of OC cycles = 1.19. Based on these values, we can conclude that there is not sufficient multicollinearity to confound the use of these three parameters.

**Fig. 3:**
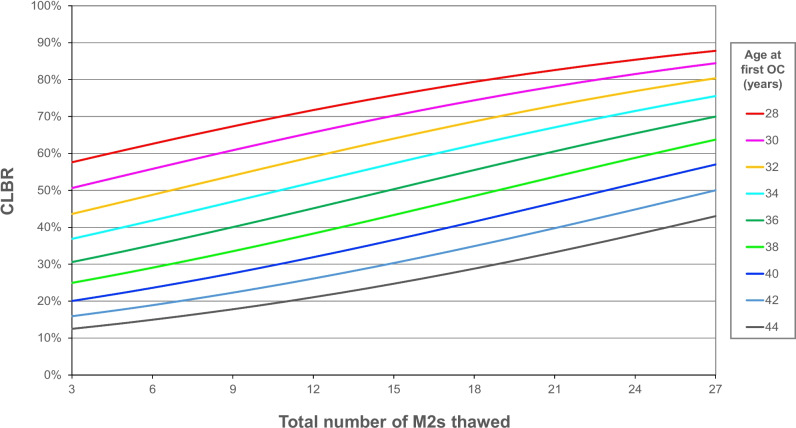
Multiple logistic regression model for cumulative live birth / ongoing pregnancy rate (CLBR) based on age at first cryopreservation and total number of metaphase II oocytes (M2s) thawed

Figure 3 shows CLBRs from our multiple logistic regression model. As expected, this model shows that CLBR is influenced by the age at OC and the number of M2s thawed.

In total, we report 313 patients with ≥ 1 live birth or ongoing pregnancy > 12 weeks from OC. These patients have a total of 370 babies or fetuses > 12 weeks. 258 patients had one live birth or fetus > 12 weeks, 50 patients had two live births or fetuses > 12 weeks (31 patients with two singletons, 19 patients with twins), and 4 patients had three live births (1 patient with 3 singletons, 2 patients with twins followed by a singleton, 1 patient with triplets) from OC. Among all patients, 26% (*n* = 188/731) have remaining cryopreserved oocytes and/or euploid/untested embryos from OC. Among those with one live birth or fetus > 12 weeks from OC, 57% (*n* = 148/258) have remaining cryopreserved oocytes and/or euploid/untested embryos from OC.

## Discussion

As use of OC increases, outcome data must be published so patients can make educated decisions about how to best employ this technology to preserve their fertility. Patients must be counselled that younger age at OC and more mature oocytes improve cumulative live birth rates. However, additional OC cycles from different months do not independently improve cumulative live birth rates. The benefit of additional OC cycles can be explained by the increased number of oocytes. Our results provide more information about the CLBR from OC based on age at OC and number of cryopreserved oocytes. This data can help patients decide how many oocytes to cryopreserve.

We provide a CLBR per patient, which is the most useful parameter when counselling OC patients. Our model, which predicts CLBR based on age at OC and total number of M2s thawed can help physicians provide personalized patient counselling. This information not only arms patients with realistic expectations, but can also help them decide whether to pursue additional OC cycles to obtain more oocytes. For example, a 34 year-old patient with 10 M2s from her first OC cycle can be counseled that cryopreserving 10 additional M2s will improve CLBR from approximately 49% to approximately 66%.

The optimal number of oocytes to cryopreserve varies for each patient. This number is not only influenced by the age at OC, but also by the patient’s ovarian reserve, desired likelihood of success, family building goals, and financial situation. Counseling must be individualized, but the predicted CLBRs from our models can help patients decide whether they would benefit from additional oocytes from additional OC cycles. Younger patients with a high number of oocytes from their first OC cycle may decide that the risks and cost of additional OC cycles outweigh the benefits.

Due to the time delay between OC and oocyte thaw, 38% of patients in this cohort underwent their first OC cycle between 2004 and 2012. OC was experimental during this time, and was not covered by employers or insurance. Due to the experimental nature and high cost of OC, patients who obtained a reasonable number of oocytes during their first OC cycle rarely pursued additional OC cycles. By contrast, patients who obtained a small number of oocytes during their first OC cycle often chose to pursue additional OC cycles to increase their chance of live birth. Now that OC is no longer considered experimental and is sometimes covered by employers or insurance, more patients with a reasonable number of oocytes from their first OC cycle are electing to pursue additional OC cycles.

This study does not provide information about the chance of achieving a second live birth from OC because most of our patients with one live birth from OC have not yet returned to use their remaining embryos. However, we report 50 patients with ≥ 2 children from OC, and the fact that 57% of patients had remaining cryopreserved oocytes and euploid/untested embryos from OC after one live birth suggests that many patients can build larger families from OC.

It is also important to note that our study includes oocytes that were slow-frozen in addition to those that were vitrified. Although our center now exclusively uses vitrification to cryopreserve oocytes, previous data from our center demonstrates that our slow-frozen oocytes performed very well [1, 11]. At our center, the decision to use vitrification alone was primarily due to laboratory efficiency, rather than improved outcomes with this cryopreservation method.

One major strength of this study is that it uses real OC data, rather than data based on IVF patients and oocyte donors. Our CLBRs for younger patients are lower than the CLBRs predicted by both Fox and Goldman.’s [12] and Maslow et al.’s [10] models. For example, Fox and Goldman’s model predicts that patients who undergo OC at age 34 and cryopreserve 6, 12, 18, and 24 M2s have CLBRs of 50%, 75%, 88%, and 94% respectively, while our model predicts respective CLBRs of 42%, 52%, 62%, and 71% for these patients. Similarly, Maslow et. al’s model predicts that women aged < 35 years have a 70% estimated live birth rate when 10 M2s are obtained from their first retrieval and a 80% predicted live birth rate when 17 M2s are obtained from their first retrieval, while our model predicts lower live birth rates for women, especially as they approach 35 years. Therefore, we suggest that OC models that rely on extrapolated data from IVF patients with normal ovarian reserve and oocyte donors be used with caution because they likely overestimate CLBRs for younger patients. We recommend using data from real OC patients for patient counseling.

This study is limited because Anti-Müllerian Hormone (AMH), Body Mass Index (BMI), and race/ethnicity were not included in our analysis and may impact the number of oocytes retrieved and live birth rates. This study is also limited by the relatively small number of OC patients who have returned for thaw. Many OC patients do not return to use their cryopreserved oocytes, and those who do return to use their cryopreserved oocytes tend to return several years later [13, 14]. Moreover, the majority of patients (72%) in this study only underwent 1 OC cycle. Larger studies are required to corroborate our findings about the effect of cycle number and to more precisely detect differences in CLBR based on age at OC and number of M2s. Moreover, this study includes patients who underwent oocyte thaw at a single high-volume urban university-affiliated institution and may not be generalizable to other OC patients. Further studies are needed from a variety center types and locations.

In conclusion, the number of cryopreserved M2s predicts CLBR, even at young ages. However, additional OC cycles do not independently improve cumulative live birth rates. This study is the first report of oocyte thaw outcomes to examine the influence of OC cycle number on CLBR, and suggests that additional OC cycles do not increase CLBR beyond increasing oocyte quantity. Our results can help OC patients estimate their CLBR and decide whether to pursue additional OC cycles to obtain more oocytes.

## Data Availability

The data included in this study is not publically available.
